# The ‘Obesity First’ approach: Redefining the future of healthcare

**DOI:** 10.1007/s12664-025-01882-5

**Published:** 2025-10-28

**Authors:** Chris J. J. Mulder, Ahmed B. Bayoumy, Azhar R. Ansari

**Affiliations:** 1 Department of Gastroenterology and Hepatology, Amsterdam University Medical Center, Vrije Universiteit Amsterdam, Amsterdam, The Netherlands; 2 Amsterdam Gastroenterology Endocrinology Metabolism Research Institute, Amsterdam, The Netherlands; 3 Department of Gastroenterology and Hepatology, Kings College Hospital, London, UK

**Keywords:** Bariatric surgery, GLP-1, Glucagon-like peptide-1 receptor agonist, Metabolic-associated steatotic liver disease, Obesity, Obesogenic environment

## Abstract

Obesity is a highly prevalent, chronic disease driven by food addiction and associated with increased premature mortality. Obesogenic environments promote unhealthy behavior, making weight management challenging. Until recently, effective pharmacological treatments were lacking. The introduction of glucagon-like peptide-1 receptor agonists (GLP-1 RAs) represents a major breakthrough in obesity care, with the potential to transform treatment strategies. Despite their efficacy, high costs (as of 2025) limit accessibility, particularly in low and middle-income regions, where parallel, unregulated use is emerging. Obesity remains under-recognized as a primary medical condition, especially in populations prone to metabolic complications, including metabolic dysfunction-associated steatotic liver disease (MASLD). Gastroenterology has historically underestimated the role of GLP-1 RAs in the past. Moving forward, the choice between GLP-1 therapy and bariatric endoscopy/surgery will become a central research focus, with treatment failures in one modality already leading to crossover. GLP-1 RAs are expected to significantly impact obesity-related comorbidities, including hypertension, dyslipidaemia, type-2 diabetes, sleep apnea, MASLD and inflammatory bowel disease (IBD). An “Obesity First” approach may reshape healthcare by addressing obesity as the primary topic cause for chronic disease. By 2035, the role of GLP-1 RAs as potential lifelong treatment will become clearer, with generic market expansion anticipated following patent expirations (China 2026; Europe 2031).

## Introduction

Obesity has emerged as the most visible global health issue, drawing public attention in ways that other chronic conditions, such as hypertension, cancer or inflammatory bowel disease (IBD), do not [1]. Recognized as a complex, relapsing and multifactorial condition, obesity is strongly linked to food addiction and increasing mortality rates [2]. Despite decades of public health campaigns encouraging healthier eating and more physical activity, lifestyle-based interventions have largely failed to curb the global rise in obesity. Over the past 40 years, obesity rates have tripled, placing a heavy strain on healthcare systems worldwide [3, 4]. Contemporary lifestyles encourage unhealthy habits, driven by the widespread availability and aggressive marketing of calorie-dense, highly palatable foods. This so-called obesogenic environment encompasses the settings and influences that promote weight gain [5]. Overeating itself can be viewed as a chronic, relapsing condition—characterized by compulsive eating and loss of control over food intake—paralleling addictions such as nicotine or alcohol dependence [6]. Until recently, treatment options were limited, leaving many individuals caught in a persistent cycle of weight gain and metabolic dysfunction.

## Epidemiology of obesity

Obesity affects individuals across all age groups and socio economic levels. According to the World Health Organization, the global prevalence of obesity has nearly tripled since 1975. By 2022, more than 2.5 billion adults were overweight, with over 890 million classified as obese. Although once predominantly seen in high-income countries, obesity rates have been rising rapidly in low and middle-income nations since the latter half of the 20th Century, driven by urbanization, economic growth and shifting dietary habits [7]. As a result, many of these countries now face a “double burden” of disease, where undernutrition and obesity coexist within the same populations [8].

## South Asian context and metabolic complications

In South Asian populations, obesity presents unique challenges. Central obesity, characterized by increased visceral adiposity, is prevalent even among individuals with a relatively low body mass index (BMI) [9]. Socio-economic factors, including rising household wealth, contribute to increased abdominal obesity across all income groups [10]. South Asians are disproportionately affected by metabolic dysfunction-associated steatotic liver disease (MASLD), a condition that affects an estimated 30% to 40% of the population [11, 12]. Children and adolescents are also increasingly affected [13]. As of 2020, an estimated 40 million children under the age of 5 were overweight or obese globally. Among school-aged children and adolescents, the prevalence has risen more than tenfold in some countries over the past four decades [14].

## The obesogenic environment and genetic susceptibility

Obesity results from the interaction between genetic predisposition and environmental factors. Traits that historically conferred survival advantages—such as efficient fat storage and heightened hunger—have become disadvantages in our modern societies [15]. Such obesogenic environments are defined by the ubiquitous availability of inexpensive, calorie-dense foods, large portion sizes and sedentary lifestyles, often exacerbated by urban designs that discourage physical activity [16]. India exemplifies how rapid urbanization, economic growth and cultural shifts are creating an obesogenic environment [17]. Traditional diets rich in whole grains, legumes, fruits and vegetables are increasingly replaced by calorie-dense, processed foods high in refined sugars and unhealthy fats. The expansion of fast-food outlets, aggressive marketing of sugary beverages and the availability of inexpensive snack foods contribute to unhealthy dietary patterns across all socio-economic strata. Physical inactivity further amplifies the problem. Urban development prioritising motorized transport over active commuting, limited access to recreational spaces and a rise in sedentary occupations contribute to declining physical activity levels, particularly among urban populations [18]. These changes have collectively driven obesity rates to epidemic proportions globally.

## Lifestyle interventions

While reduced-calorie diets combined with physical activity and behavioral support can lead to modest yet clinically significant weight loss (typically 5% to 10% of initial body weight), their long-term effectiveness is often disappointing [19]. Lifestyle modification has traditionally been the cornerstone of obesity treatment, encompassing structured programs aimed at dietary change, increased physical activity and behavioral therapy [19]. However, despite widespread implementation over the past 30 to 40 years, such interventions have failed to halt the global rise in obesity.

## Pharmacotherapy developments from 1970 onward

Pharmacological treatment for obesity may be more effective when combined with lifestyle interventions and delivered as part of a structured, multidisciplinary care approach. Crucially, medication should not be seen as a short-term fix, but rather as a component of long-term chronic disease management. Historically, pharmacologic strategies for weight loss have faced numerous setbacks [20]. For example, fenfluramine (Ponderal^®^), widely used between the 1970s and 1990s as an appetite suppressant, was withdrawn from the market due to its association with pulmonary hypertension [21]. Current pharmacologic options include the combination of naltrexone and bupropion, which targets appetite and reward pathways but carries an increased risk of adverse effects [22]. Orlistat, used alone or with metformin, works by inhibiting fat absorption in the intestine [23]. While these treatments can achieve moderate weight loss—typically around 5% to10%—they are often limited by side effects and issues with patient adherence. Therefore, their use requires a careful, individualized assessment of risks and benefits [23].

## Emergence of GLP-1 receptor agonists

The development of glucagon-like peptide-1 (GLP-1) receptor agonists marks a major shift in obesity treatment [24]. These agents, initially developed for type-2 diabetes management, mimic endogenous GLP-1, enhancing satiety and reducing food intake. Liraglutide was among the first GLP-1 analogues approved for obesity treatment [6]. More recently, semaglutide and tirzepatide have demonstrated superior efficacy, with tirzepatide showing greater weight reduction in clinical trials [25, 26]. Additionally, GLP-1 receptor agonists provide secondary benefits, including improvements in metabolic parameters, cardiovascular outcomes and chronic kidney disease [27]. Common side effects are primarily gastrointestinal and include nausea, vomiting and diarrhea, particularly during dose titration [28]. GLP-1 therapies should be used cautiously in patients with a history of pancreatitis or gallbladder disease [29].

## Safety considerations and drug interactions with GLP-1 receptor agonists in clinical practice

Common side effects such as decreased appetite, dyspepsia and belching are noted in the patient information leaflet and are generally expected with GLP-1 receptor agonist therapy. However, delayed gastric emptying can lead to significant reflux symptoms, for which appropriate dosing of proton pump inhibitors (PPIs) is recommended. In cases of constipation, early intervention with macrogol and adequate fluid intake should be advised [30]. Clinicians should also be aware of the potential development of cholelithiasis and cholecystitis, with or without pancreatitis [29]. Notably, significant alterations in thiopurine metabolite levels have been reported in an IBD patient treated with tirzepatide [31]. While co-administration of oral semaglutide did not affect the pharmacokinetics of ethinylestradiol or levonorgestrel, nor the exposure of lisinopril, warfarin or digoxin, isolated cases of reduced international normalized ratio (INR) have been reported with concurrent use of semaglutide and acenocoumarol [32]. Therefore, in patients using warfarin or other coumarin derivatives, additional INR monitoring is recommended upon initiation of semaglutide [33]. Emerging case reports also suggest a possible increase in miscarriage rates, raising concerns that some patients may continue GLP-1 therapy during pregnancy despite the lack of safety data [34]. As research and development in this field progress, we can anticipate the emergence of new GLP-1 formulations that may target additional pathways and lead to different, more frequent or potentially fewer side effects.

## Market dynamics and access considerations

Despite their efficacy, GLP-1 therapies remain so far costly, limiting accessibility, particularly in low and middle-income countries [35]. However, growing demand has fuelled parallel markets, with individuals in high-income groups purchasing GLP-1 medications through unofficial channels. Shortly, patients with enough money do not wait for compensation; they usually do not have diabetes mellitus type 2 (DM2), often have a body mass index (BMI) of around 28-30 and rarely over 35. They just want to use it. There are no figures about this parallel use. Market trends suggest that generic formulations—particularly from Indian, Chinese and Brazilian manufacturers—are expected to expand access significantly following patent expirations between 2026 and 2031 [36].

## Cost-effectiveness and bariatric interventions

Bariatric surgery remains a cost-effective intervention for individuals with severe obesity, yet the growing adoption of GLP-1 receptor agonists is transforming current obesity management strategies [37]. Endoscopic bariatric procedures are increasingly recognized as a valuable intermediary option—either as a bridge to surgery or as a complement to pharmacotherapy. However, direct cost comparisons between GLP-1 therapies and various endoscopic techniques are currently lacking. Important questions remain about the optimal sequencing and integration of long-term—or potentially lifelong—pharmacotherapy, endoscopic interventions, and surgical approaches. Although GLP-1 therapies may seem more affordable in the short -term, their cumulative costs can surpass those of bariatric surgery within one to two years, particularly in light of the common weight regain observed after treatment cessation [38].

## Obesity First: a paradigm shift in chronic disease management

Obesity is a key driver of chronic conditions, including hypertension, type-2 diabetes, MASLD, cardiovascular disease, musculoskeletal disorders and potentially inflammatory bowel disease. Clinical care often remains fragmented, treating complications in isolation rather than addressing obesity as the root cause. A so-called ‘Obesity First’ approach advocates for proactive obesity treatment to prevent downstream complications. GLP-1 receptor agonists exemplify this paradigm, offering substantial efficacy in weight loss and metabolic improvement. As accessibility increases, integrating GLP-1 therapies into primary care could redefine chronic disease management. Further research is essential to evaluate long-term GLP-1 use, sustainability of weight loss, cost-effectiveness and the role of newer agents such as survodutide, pemvidutide, retatrutide and orforglipron.

## GLP-1 receptor agonists in gastroenterology: Opportunities beyond obesity

It is crucial that gastroenterologists take a proactive role in GLP-1 research, especially as these agents are increasingly being prescribed—whether or not they are currently reimbursed by health insurers. Although the high cost of GLP-1 receptor agonists remains a significant barrier for many healthcare systems, the emergence of generic formulations is expected to improve affordability. Evidence suggests that GLP-1 therapies may reduce hepatic fat accumulation, offering potential benefit for patients with MASLD [39]. The new European Association for the Study of the Liver (EASL) guidelines cautiously but clearly acknowledge a role for GLP-1 in the management of MASLD [40]. Further research in this area is essential. In the UK, GLP-1 is being explored for its potential benefits in individuals with alcohol use disorder [41]. Notably, a 2006 case report already indicated that the incretin mimetic exenatide could reduce hepatic fat accumulation, hinting at a future therapeutic role in MASLD [42]. Beyond liver disease, GLP-1 receptor agonists offer a promising avenue for drug repurposing in inflammatory conditions such as IBD [43–45]. Emerging observational studies suggest that GLP-1 therapies may lead to improved clinical outcomes in IBD. Comprehensive evaluations comparing GLP-1 therapies with bariatric endoscopy, intragastric balloon treatment and surgical interventions are needed. These comparisons are particularly important for high-risk surgical candidates or patients seeking non-surgical alternatives.

Conclusion, the global obesity epidemic demands innovative, multifaceted solutions. GLP-1 receptor agonists represent a promising addition to the therapeutic arsenal, with the potential to transform obesity management alongside lifestyle interventions and bariatric procedures. This trajectory mirrors earlier pharmaceutical innovations, such as PPIs and statins, which transformed clinical practice once affordability improved. As GLP-1 therapies become more accessible, their integration into primary care will likely redefine chronic disease management. Given obesity’s role in elevating cancer mortality risk and accelerating chronic disease, early intervention is critical. The foundational work of early GLP-1 researchers, including John Eng and Jean-Pierre Raufman in the 1980–1990s, underscores the importance of visionary science [46]. Their major contribution now shape what may become one of the most transformative shifts in modern medicine. The “Obesity First” model provides a coherent framework for preventing and mitigating chronic disease. As generics expand access and ongoing research clarifies long-term outcomes, healthcare systems will adapt to prioritise obesity treatment, ultimately improving population health and quality of life worldwide.

## Declarations

### Disclaimer

The authors are solely responsible for the data and the contents of the paper. In no way, the Honorary Editor-in-Chief, Editorial Board Members, the Indian Society of Gastroenterology or the printer/publishers are responsible for the results/findings and content of this article.
